# Expression of COX-2, CD44v6 and CD147 and Relationship with Invasion and Lymph Node Metastasis in Hypopharyngeal Squamous Cell Carcinoma

**DOI:** 10.1371/journal.pone.0071048

**Published:** 2013-09-03

**Authors:** Qing Yang, Yehai Liu, Yang Huang, Dake Huang, Yifan Li, Jing Wu, Maoli Duan

**Affiliations:** 1 Department of Otorhinolaryngology-Head and Neck Surgery, the First Affiliated Hospital of Anhui Medical University, Hefei, Anhui, PR China; 2 Department of Microbiology and Parasitology, Anhui Medical University, Hefei, Anhui, PR China; 3 Department of clinical science, intervention and technology, Karolonska Institute, Stockholm, Sweden; Chinese Academy of Medical Sciences, China

## Abstract

To assess the expression of COX-2,CD44v6 and CD147 in hypopharyngeal squamous cell carcinomas and the three biomarkers correlation with tumor invasion and lymph node metastasis of Chinese people. 101 cases of surgically excised primary tumor were included in this study, and 40 tissues of epithelium adjacent to carcinoma were used as controls. We characterized the immunohistochemical expression of COX-2, CD44v6, and CD147 in141 formalin-fixed, paraffin-embedded tissues, and measured the mean optical density (OD) of the positive area to identify the expression of the three bio-markers and relationship with tumor invasion and lymph node metastasis. Our study demonstrates that the expression of the COX-2 and CD147 were significantly increased in carcinoma tissues compared to the epithelium adjacent to carcinoma. We also observed that the expression of COX-2, CD44v6, and CD147 were significantly associated with T classification, lymph node metastasis and clinical stage. There was strong significant correlation among the three biomarkers as well. Additionally, we indicated that recurrence and ≥P_50_ level of COX-2 expression had an independent prognostic effect on prognosis. In conclusion, the three biomarkers play important roles in tumor invasion and lymph node metastases and might be valuable indicators of tumor metastasis in hypopharyngeal squamous cell carcinoma.

## Introduction

Hypopharyngeal squamous cell carcinoma, a malignant neoplasm arising from the mucosa of the upper aerodigestive tract, is one of the most aggressive cancers in the head and neck area. Over 50% of the patients with hypopharyngeal squamous cell carcinoma have reached stage IV at presentation because of lacks of symptoms. Even after multimodality therapy 20% of the patients have residual disease, and recurrences tend to appear in the first year and 50% of first recurrences include metastasis. Eventually, 64% of patients die of the cancer [1]. Furthermore, patients with invasion and metastasis to surrounding tissues in the early stages, usually have an unfavorable prognosis [2]. Although combined modality treatments such as chemotherapy, radiation and improved surgical techniques have been applied in clinic, these have not translated into significant improvements in survival [3]. Thus, it is necessary to find novel cancer-related molecules for diagnosis and consequently to improve prognosis of hypopharyngeal carcinoma.

Over the last decade, it has become clear that there are a large number of biological markers associated with invasion and metastasis of hypopharyngeal carcinoma. Cyclooxygenase-2 (COX-2), one of the cyclooxygenase enzymes key to prostaglandin biosynthesis, whose expression is dramatically up-regulated after inflammation and tumors stimulation, is rapidly induced by certain growth factors, such as inflammatory cytokines, tumor promoters and oncogenes [4]. Since the overexpression of COX-2 was reported in colorectal cancer [5], several studies have shown its overexpression in other types of cancer, especially in epithelial cancers, for example, breast, prostate and lung cancer [6]–[8]. This suggests that COX-2 may play an important role in tumor development and progression. Meanwhile, some studies have indicated the overexpression of COX-2 occurs in patients with head and neck cancer [9]–[10]. CD44v6, a variant isoformof CD44, regulates tumor invasion and metastasis formation, has been shown as a protein marker for metastatic behavior in breast, colorectal and gastric cancers [11]–[13]. These characteristics have made CD44v6 an attractive factor for the research of metastasis of hypopharyngeal carcinoma. CD147, also known as extracellular matrix metalloproteinase inducer (EMMPRIN), has been identified as a tumor-cell membrane protein that stimulates matrix metalloproteinase (MMP) production in stromal fibroblasts [14]. As a transmembrane glycoprotein of the immunoglobulin superfamily, CD147 is overexpressed in various tumor cells including those in head and neck carcinoma [15], and is also known to promote tumor invasion and lymph node metastasis [15]–[16].

Our aim is to evaluate the expression levels of COX-2, CD44v6 and CD147 in hypopharyngeal squamous cell carcinoma, and to examine relationship between these three bio-markers with tumor invasion and lymph node metastasis. Furthermore, we analyze the correlation among them and figure out if they can be indicated the tumor invasion and lymph node metastasis.

## Methods

### Cases

The study protocol was approved by the Ethics Committee of the First Affiliated Hospital of Anhui Medical University, all procedures were performed under the Helsinki declaration, and written consent was obtained from all patients.

101 cases of surgically excised primary tumor were included in this study. Paraffin-embedded specimens of 71 patients with hypopharyngeal squamous cell carcinoma were obtained from the Department of Pathology of the first Hospital Affiliated to Anhui Medical University. These patients were treated at the department of Otorhinolaryngology, Head and Neck Surgery, during January 2004–June 2010. The other 40 cancer tissues were obtained intra-operatively from patients who were operated in the hospital during July 2010 to May 2011. Meanwhile, 40 tissues of epithelium adjacent to carcinoma obtained by the same way were used for comparison.

No patient had received any prior therapy, such as radio- or chemotherapy. All hematoxylin-eosin-stained sections were reviewed, the quality of the material was checked, and the best section from each specimen was selected. All tumors were classified histologically according to the classification by WHO, TNM and clinical stages were identified by IUCC system.

### Immunohistochemistry study

All sections were deparaffinized in xylene, sequentially rehydrated in alcohol, and washed in phosphate-buffered saline. The sections were heated twice in a microwave oven for 5 min in citrate buffer (pH 6.0) for antigen retrieval. COX-2 monoclonal antibody (1∶200; Santa Cruz Biotechnology Inc., Santa Cruz, CA), CD44v6 (ready to use; Zhongshan biologic and technical company, Beijing, China) and CD147 (1∶150; Santa Cruz Biotechnology Inc., Santa Cruz, CA) were used as the primary antibody. Briefly, the incubation was at 4°C overnight, followed by washing with PBS. The sections were incubated with secondary antibody for 30 min at room temperature. Signals were developed with 3,3′-diaminobenzine (DAB) for 2 min and counterstained with hematoxylin.

### Quantitative analysis

The immunostaining densities of COX-2, CD44v6 and CD147 in hypopharyngeal carcinoma were quantitatively assessed with the Image-pro plus6.0 (Media Cybernetics, USA). In brief, sections were placed on a microscope (Olympus CX21, Japan), and images were transferred via a digital camera (Nikon 80i, Japan) to a computer. The mean optical density (OD) of the positive areas was measured. The results were expressed as the exact value of the relative optical density units.

### Statistical analysis

The SPSS software (version 17.0., SPSS Inc., Chicago, IL, USA) was used in this study. *t* test was applied to compare variables. Pearson correlations between three bio-markers were estimated. Survival and recurrence were assessed with the Kaplan-Meier method and comparisons between subgroups were conducted using the log rank test. Cox's proportional hazards regressions were used to find out the significant predictors for the survival time. Significant was at the level of *P*<0.05.

## Results

### Clinicopathologic characteristics data

The clinicopathologic characteristics of the patients in the study are as follow: the mean age of the patients was 60.73 years, and the range was from 42 to 78. Of the patients, 99 of the patients were males and 2 were females. 59 of those (58%) presented with lymph node metastasis at diagnosis (Table 1).

**Table 1:** Clinicopathologic characteristics data of patients.

	N(%)/mean
**Gender**	
male	99 (98%)
female	2 ( 2%)
**Age (years)**	60.73
**T stage**	
T1+T2	43 (43%)
T3+T4	58 (57%)
**Lymph node**	
Positive	59 (58%)
Negative	42 (42%)
**Clinical grade**	
I+II	22 (22%)
III+IV	79 (78%)
**Histologic grade**	
Well	14 (14%)
Moderate	51 (50%)
Poorly	36 (36%)
**Total**	101 (100%)

### Expression of COX-2, CD44v6 and CD147

**Figure 1:**
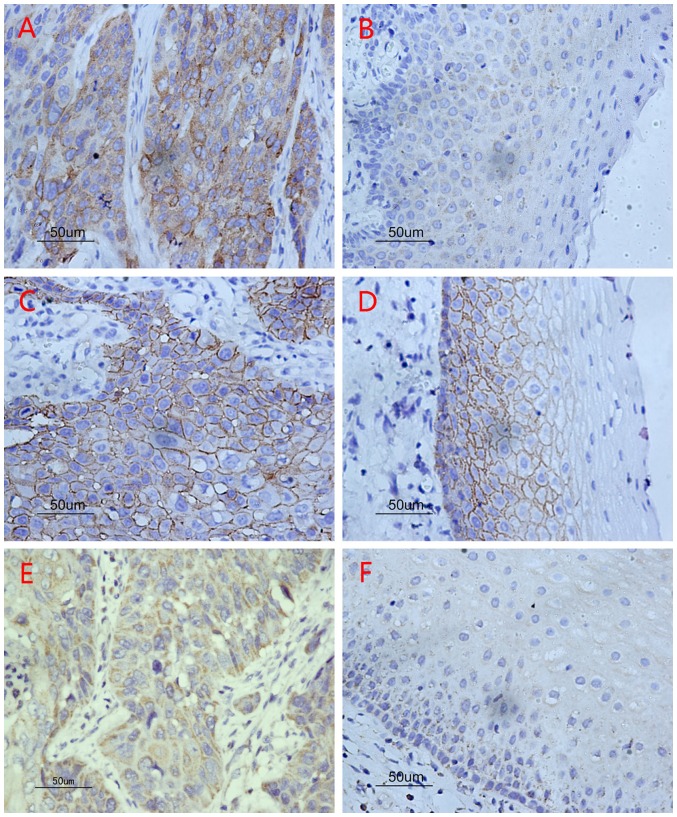
The three bio-markers expression by immunohistochemistry. (A) High levels of COX-2 expression in hypopharyngeal squamous cell carcinoma: diffuse cytoplasmic staining. (B) Low levels of COX-2 expression in epithelium adjacent to carcinoma. (C) High levels of CD44v6 expression in hypopharyngeal squamous cell carcinoma: cell membrane staining. (D) High levels of CD44v6 expression in epithelium adjacent to carcinoma as well as staining in carcinoma tissues. (E) High levels of CD147 expression in hypopharyngeal squamous cell carcinoma: diffuse cytoplasmic staining. (F) Low level of CD147 expression in epithelium adjacent to carcinoma.

**Figure 2:**
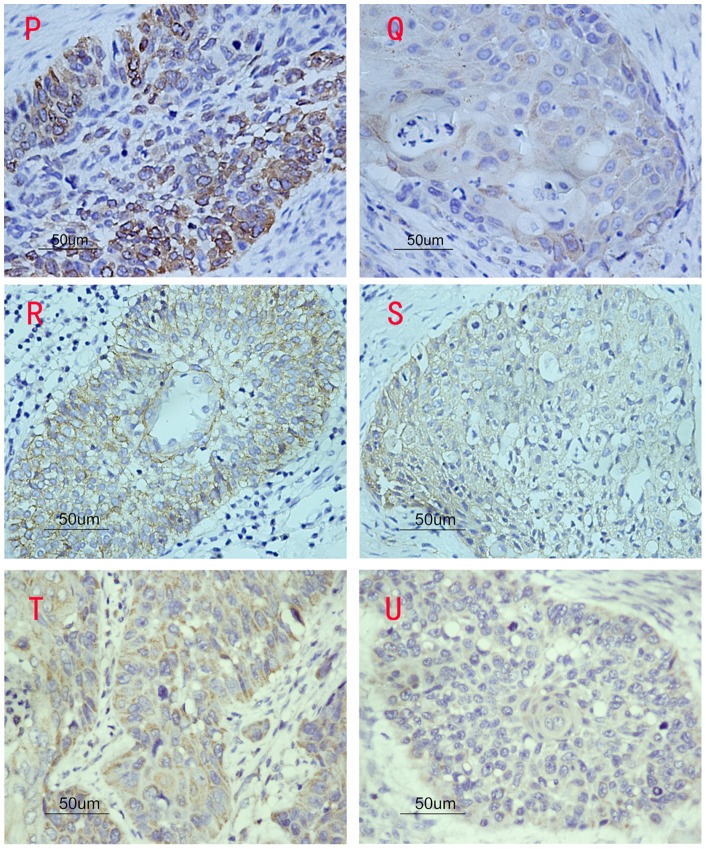
The three bio-markers expression in tumor tissues with and without lymph node metastasis. (P) High levels of COX-2 expression in tumor tissue with lymph node metastasis. (Q) Low levels of COX-2 expression in tumor tissue without lymph node metastasis. (R) High levels of CD44v6 expression in tumor tissue with lymph node metastasis. (S) Low levels of CD44v6 expression in tumor tissue without lymph node metastasis. (T) High levels of CD147 expression in tumor tissue with lymph node metastasis. (U) Low levels of CD147 expression in tumor tissue without lymph node metastasis.

**Figure 3:**
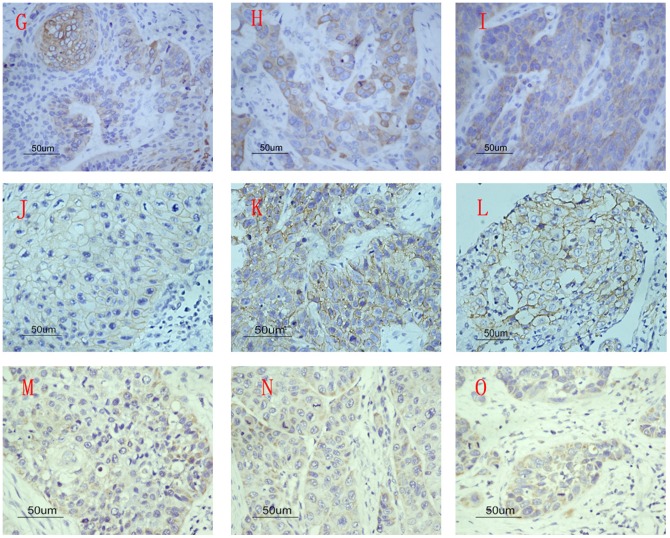
The three bio-markers expression in well, moderate and pooly differentiated tumor tissues. (G) COX-2 expression in well differentiated tumor tissues. (H) COX-2 expression in moderate differentiated tumor tissues. (I) COX-2 expression in poorly differentiated tumor tissues. (J) CD44v6 expression in well differentiated tumor tissues. (K) CD44v6 expression in moderate differentiated tumor tissues. (L) CD44v6 expression in poorly differentiated tumor tissues. (M) CD147 expression in well differentiated tumor tissues. (N) CD147 expression in moderate differentiated tumor tissues. (O) CD147 expression in poorly differentiated tumor tissues.

In this study, diffuse cytoplasmic staining for COX-2 appeared in almost all carcinoma tissues and 70.0% (28/40) of adjacent normal epithelium tissues. However, normal epithelium presented weaker COX-2 staining (part A and B, Figure 1). The mean optical density (ODs) of COX-2 in carcinoma tissues and adjacent normal epithelium tissues were 0.25±0.11 and 0.08±0.06, respectively (Table 2, Table 3). COX-2 expressed in carcinoma tissues was significantly higher than that in adjacent normal tissues (*P*<0.001). Moreover, increased COX-2 expression was strongly associated with lymph node metastasis (part P and Q, Figure 2; *P*<0.001), T classification (*P*<0.001) and clinical stage (*P*<0.001). No correlation was found between COX-2 and histological grades (part G, H and I, Figure 3; *P* = 0.956).

**Table 2:** Correlation of clinicopathologic characteristics of the patients with immunohistochemistry results.

Variable	Case	COX-2	CD44v6	CD147
	N (%)	ODs *t/F*	ODs *t/F*	ODs *t/F*
**T stage**				
T1+T2	43(43)	0.20±0.08 –4.085*	0.18±0.06 –2.597*	0.14±0.07 –3.365*
T3+T4	58(57)	0.28±0.11	0.21±0.07	0.19±0.07
**Lymph node**				
Positive	59(58)	0.31±0.05 –10.559*	0.24±0.05 –10.771*	0.23±0.03 –27.582*
Negative	42(42)	0.15±0.09	0.14±0.04	0.09±0.02
**Clinical stage**				
I+II	22(22)	0.14±0.06 –5.4289*	0.12±0.02 –7.0419*	0.08±0.02 –8.0459*
III+IV	79(78)	0.27±0.10	0.22±0.06	0.19±0.06
**Histologic grade**				
Well	14(14)	0.24±0.11 0.046	0.19±0.07 0.210	0.16±0.08 0.464
Moderate	51(50)	0.25±0.11	0.20±0.07	0.18±0.07
Poorly	36(36)	0.25±0.10	0.21±0.05	0.17±0.07
**Total cases**	101(100)	0.25±0.11	0.19±0.07	0.17±0.07

* *P*<0.001.

**Table 3:** The three bio-markers expression in carcinoma and adjacent epithelium.

Variable	Case	COX-2	CD44v6	CD147
	N (%)	ODs *t*	ODs *t*	ODs *t*
**Carcinoma tissues**	101(72)	0.25±0.11 9.004*	0.19±0.07 –0.429	0.17±0.07 −9.450*
**Epithelium tissues adjacent to carcinoma**	40(28)	0.08±0.06	0.20±0.04	0.05±0.06

* *P*<0.001.

CD44v6 was expressed in all carcinoma tissues, mainly in the cell membrane, and staining intensity for cells in adjacent epithelium issues appeared similar to that in carcinomas (part C and D, Figure 1). There was no significant difference between carcinoma tissues and tissues adjacent to carcinoma (*P* = 0.668). However, CD44v6 expression was strongly correlated with lymph node metastasis (part R and S, Figure 2; *P*<0.001), T classification (*P*<0.001) and clinical stage (*P*<0.001), but not correlated to histological grade (part J, K and L, Figure 3; *P* = 0.811).

CD147 was mainly expressed in cell membranous and cytoplasmic tissues, and also in all carcinoma tissues and adjacent normal epithelium (47.5%, 19/40) (part E and F, Figure 1). The mean OD measures indicate that CD147 expression in hypopharyngeal squamous cell carcinoma was significantly higher than that in adjacent epithelium to carcinoma (*P*<0.001). Although no significant difference was observed between the grade of differentiation of the tumors (part M, N and O, Figure 3; *P* = 0.630), the level of CD44v6 expression was significantly associated with the incidence of lymph node metastasis (part T and U, Figure 2; *P*<0.001), T classification (*P* = 0.001) and clinical stage (*P*<0.001).

### Correlation analysis

Pearson correlation analyses indicate a strong significant correlation between COX-2 and CD147 (*r* = 0.774, *P*<0.001). And there were correlations between COX-2 and CD44v6 (*r* = 0.473, *P*<0.001), as well as between CD44v6 and CD147(*r* = 0.475, *P*<0.001).

### Survival analysis

Twenty-one patients were lost during follow-up in this study, and 37 patients suffered from a recurrence. Survival analyses among 80 patients show that 1-year survival rate was 66.25%, 3-year survival rate 16.25% and 5-year survival 7.5%.

**Figure 4:**
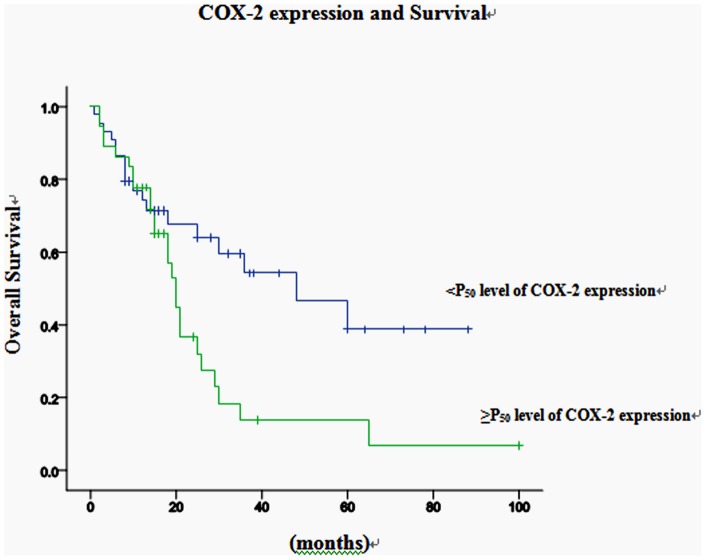
Kaplan-Meier curve for the overall survival of the patients with hypopharyngeal carcinoma, according to the expression of P_50_ of COX-2.

**Figure 5:**
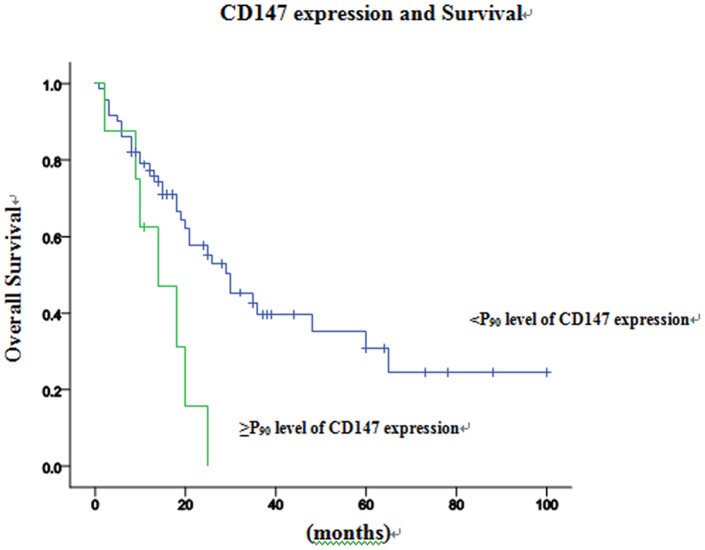
Kaplan-Meier curve for the overall survival of the patients with hypopharyngeal carcinoma, according to the expression of P_90_ of CD147.

Results indicate that mortality of ≥P_50_ level of COX-2 expression was higher than that of < P_50_ level of COX-2 expression, and the difference was significant (Table 4). Survival time is significantly correlated with≥P_75_ level of COX-2 expression, ≥P_85_ level of COX-2 expression and ≥P_90_ level of COX-2 expression (*P*<0.05). Meanwhile, it is significantly associated with ≥P_90_ level of CD147 expression (*P*<0.05) as well. But there is no relationship between survival time and the level of CD44v6 expression. On the basis of the above results, ≥P_50_ level of COX-2 expression and ≥P_90_ level of CD147 expression were classified as the expression of COX-2 and CD147 in hypopharyngeal squamous cell carcinoma for Cox's proportional hazard model analysis. Univariate analysis indicated that recurrence, ≥P_50_ level of COX-2 expression and ≥P_90_ level of CD147 expression were associated significantly with a worse prognosis (Figure 4 and 5). Neither clinical stages nor node metastasis had any significant association with survival. Multivariate Cox's proportional hazard model analysis indicates that recurrence and ≥P_50_ level of COX-2 expression had an independent prognostic effect on prognosis (*P*<0.05; Table 5).

**Table 4:** Kaplan-Meier survival analysis by level of the three bio-markers expression.

Variable	≥P_50_	≥P_75_	≥P_85_	≥P_90_
	n_1_ n (%) *χ^2^*	n_1_ n (%) *χ^2^*	n_1_ n (%) *χ^2^*	n_1_ n (%) *χ^2^*
**COX-2**	39 26(66.7) 6.104*9	21 17(80.9) 6.870*9	12 11(91.7) 8.028*9	7 6(85.7) 4.725*9
**CD44v6**	41 24(58.8) 0.752	21 12(57.1) 0.068	12 8(66.7) 0.433	7 5(71.4) 0.313
**CD147**	40 25(62.5) 1.629	17 11(64.7) 0.059	12 9(75.0) 0.520	8 7(87.5) 7.059*9

* log rank *P*<0.05.

n_1_ is the number of total patients of relative percentile;

n (%) is the number of dead patients of relative percentile (n/n_1_*100%).

**Table 5:** Cox proportional hazards regression models in estimating cancer progression.

Variables	Relative risk (95%confidence interval)	*P*
**Univariate analysis**		
Recurrence(no/yes)	3.372(1.784–6.376)	0.000
≥P_50_ level of COX-2 expression	2.143(1.147–4.003)	0.017
≥P_90_ level of CD147 expression	2.913(1.261–6.729)	0.012
**Multivariate analysis**		
Recurrence(no/yes)	3.508(1.850–6.651)	0.000
≥P_50_ level of COX-2 expression	2.158(1.146–4.062)	0.017

* *P*<0.001.

## Discussion

In this study, we investigated the prognostic significance of expression of three bio-markers (COX-2, CD44v6 and CD147) in hypopharyngeal squamous cell carcinoma and epithelium adjacent to carcinoma. The results showed that the expressions of the three bio-markers were significantly associated with tumor invasion and lymph node metastasis.

Regarding previous reports, COX-2 had an important role in invasion and metastasis of head and neck squamous cell carcinoma by a variety of pathways [17]–[18].To produce a marked effect, COX-2 was mediated by a series of molecules, for instance, CD44, matrix metalloproteinases and VEGF, lead to promote tumor angiogenesis and invasion, even had contribution to cell proliferation and apoptosis [19]. In the present study, we suggested that COX-2 expression was significantly higher in the carcinoma samples than in the adjacent to carcinoma samples, and it was also strongly associated with the presence of lymph node metastases and tumor invasion. Furthermore, no correlation was found between COX-2 expression and histological grades. This result is generally consistent with previous reports (9).

CD44v6 as a kind of CD44 variant isoforms, is regarded to be responsible for tumor lymphangiogenesis and lymph node metastasis. On the one hand, studies indicated that the overexpression of CD44v6 in squamous cell carcinoma is associated with lymph node metastasis derived from skin, lung, gastric cancers as well as head and neck [20]–[22]. On the other hand, CD44v6 was considered as a factor of “down-regulation” because CD44v6 expression is weak or absent in certain samples of squamous cell carcinoma [23], [24]. Therefore, the clinical significance of CD44v6 in squamous cell carcinoma remains controversial. In this study, results show that increased CD44v6 expression was strongly associated with lymph node metastasis. Our finding is similar to Guler et al. who found that the expression of CD44v6 was an indicator of malignant potential of the tumors in squamous cell carcinoma of the larynx [25]. This study also shows that the higher the grade of T classification, the higher the level of CD44v6 expression, and the difference is statistically significant. Besides, in our study, the level of CD44v6 expression in squamous cell carcinoma was comparable to that in normal squamous epithelium, which was similar with the result of Mack's [26].

CD147, also known as EMMPRIN, plays a crucial role in tumor progression, invasion and metastasis in head and neck squamous cell carcinoma [27]. This study has demonstrated that the level of CD147 expression in hypopharyngeal carcinoma was significantly higher than that in epithelium adjacent to carcinoma. It also found that CD147 expression was significantly correlated with T classification, clinical stage and lymph node status. These findings are the same as in previous reports [16], [28].

Lymph node metastasis is one of the important factors for outcome of hypopharyngeal cancer patients. There are several pathways in process of lymphatic metastasis of tumor cells, such as tumor lymphangiogenesis, migration, adhesion and proliferarion. Previous study revealed that COX-2 overexpression stimulated VEGF-C, a biomarker considered to be responsible for tumor lymphangiogenesis, up-regulated and induced the growth of new lymphatic vessels, which might be the first step for spreading of tumor cells to the lymph nodes [9]. It has also been reported that CD44v6 was associated with tumor growth and lymph node metastasis. But the relationship between CD44v6 and tumor invasion remains a controversial issue in fact. Sikorska et al [29] reported that they found CD44v6 had no impact on tumor progression and metastasis. However, the mechanism of CD44v6 in the process of tumor metastasis has remained obscure, and CD44v6 could help tumor cells to escape identification and killing from immune system to promote lymph node metastasis [30]. CD147 can stimulate tumor cells in synthesis of MMPs and mediate the degradation of the extracellular matrix, playing an important role in tumor invasion and metastasis. CD147 can also stimulate VEGF expression to promote tumor angiogenesis by up-regulating the urokinase-type plasminogen activator system. As well as, it mediates a series of tumor promoting molecular events to facilitate tumor invasion and metastasis [31].

Our findings demonstrate a significant correlation of COX-2 and CD147 with survival, which suggests that COX-2 and CD147 are potential bio-markers for prognosis in head and neck squamous cell carcinoma. Lymph node status, T classification or clinical stage did not show any significant association with survival time, although there is a trend towards correlation of the presence of lymph nodes at the time of diagnosis with worse survival from the clinical standpoint.

In our study, we come to the conclusion that survival time has significant correlation with ≥P_75_ level of COX-2 expression and ≥P_90_ level of CD147 expression by measuring the optical density of positive areas. In addition, we select effective percentile values to reflect the correlation between the bio-markers expression and prognosis which provides us with a novel way to predict the progression and prognosis of the tumor.

There were some limitations in our study. Firstly, further study should focus on the relationship of the three bio-marker pathways with tumor growth, metastasis and survival, which could help to find better targets for therapeutic measures. Secondly, all the patients were Chinese Han population, and only 2 patients were females which may be poorly representative of the whole population. Studies on multi-ethnic populations may clarify the function of the three bio-markers in hypopharyngeal squamous cell carcinoma.
